# Predicting Haul Truck Travel Times in Underground Mines

**DOI:** 10.1007/s42461-025-01293-2

**Published:** 2025-07-04

**Authors:** Victor Simon, Robert Pellerin, Michel Gamache

**Affiliations:** 1 Department of Mathematics and Industrial Engineering, Polytechnique Montreal, 2500 Chemin de Polytechnique, H3T 1J4 Montreal, QC Canada; 2 CIRRELT (Interuniversity Research Centre on Enterprise Networks, Logistics and Transportation), 3rd floor, office 3520, 2920 Chemin de la Tour, Pavillon André-Aisenstadt, Campus of the University of Montreal, H3T 1J4 Montreal, QC Canada; 3 GERAD (Group for Study and Research in Decision Analysis), 4th floor, office 4465, 2920 Chemin de la Tour, Pavillon André-Aisenstadt, Campus of the University of Montreal, H3T 1N8 Montreal, QC Canada

**Keywords:** Underground mining, Travel time prediction, Haul trucks, Machine learning, Stacking integrated model, Autonomous driving

## Abstract

Accurately predicting haul truck (HT) travel times (TT) in underground mines is essential for enhancing operational planning, as it allows planners to forecast extraction rates at each work face, minimize queue-related downtime, and ultimately increase productivity. However, in underground environments where GPS signals are unavailable, beacon-based locating systems have not yet been utilized for this predictive purpose. This study addresses that gap by introducing a machine learning approach for HT TT prediction that relies exclusively on beacon detection data, thus eliminating the need for traditional telemetry. The proposed method combines three route-segmentation strategies—full-route, short-segment, and major-segment predictions—with Gaussian mixture models, long short-term memory networks, and a stacking ensemble. Validated on two underground mines, it outperformed industry benchmarks, reducing prediction error by up to 34% on ascending routes and 18% on descending routes while achieving even greater precision for autonomous HTs. It showcases the untapped potential of beacon-based location systems for predictive applications, supporting mine planners.

## Introduction

A survey published by the audit and consulting firm EY in October 2024 highlights the rising capital-related risks and macroeconomic challenges facing the mining sector in 2025 [1]. These pressures drive industry stakeholders to improve productivity and reduce operating costs, making short-term planning, real-time scheduling, and dispatching systems increasingly critical. Short-term (or operational) planning refers to the precise scheduling of upcoming operations—often on a per-shift basis [2, 3]. This scheduling process is flexible and frequently updated with real-time data to curb non-value-adding activities such as wait times, thereby enhancing overall extraction productivity [4]. Effective short-term planning can also improve workplace safety and reduce per-ton fuel consumption.

A key challenge within this planning framework is the accurate prediction of haul truck (HT) travel times (TT), which critically impacts productivity and operational costs [5, 6]. In underground mines, this task becomes far more complex than on the surface, given limited visibility, narrow roadways, mixed traffic with utility vehicles and pedestrians, and frequent route adjustments [7, 8]. Historically, collecting reliable TT data underground has been especially challenging due to the lack of GPS coverage. However, new “mine 4.0” technologies—ranging from improved wireless communication to beacon-based locating systems—now enable the large-scale gathering of trip-related data [9]. Despite these advances, many underground mining operations have yet to leverage beacon data specifically for TT prediction, signaling an opportunity to refine short-term planning through more precise TT forecasts.

Exploiting beacon data for precise HT TT prediction would provide significant operational benefits. Before each shift, planners would gain improved insights into expected traffic conditions at various ramp sections, facilitating better truck fleet allocation, optimized scheduling, and prevention of bottlenecks on single-lane ramps. During shifts, real-time TT prediction would enable the proactive detection of potential head-on encounters or reverse maneuvers. It would allow early traffic management interventions through a real-time fleet management system, such as adjusted departure times, strategic bay usage, or speed modulation. Collectively, these improvements promise higher equipment utilization, enhanced operator safety, and reduced idle fuel consumption.

Given (i) the necessity of accurate HT TT prediction for robust short-term planning, (ii) the inherent complexity of this task in underground mining, and (iii) the availability of emerging beacon-based data sources, our study **aims to develop a high-performance prediction model for HT TTs using only variables that are reliably accessible before each shift**. The proposed approach is tested on two distinct mining sites to demonstrate its performance.

The remainder of this paper is structured as follows. Section 2 reviews the existing literature on HT TT prediction in both surface and underground contexts. Section 3 details the data collection process at the underground mining site. Section 4 describes the development of our integrated TT prediction model. Section 5 evaluates the model’s performance. Section 6 explores the model’s generalization to a second mining site. Finally, Section 7 concludes and highlights avenues for future work.

## Literature Review

A systematic literature review was conducted to address the prediction of HT TTs in mines, retaining only 15 documents (see Appendix 1). These documents are synthesized in the following section and an overview can be found in Table 5, Appendix 1. The results will be analyzed in three main categories to distinguish existing approaches: machine learning (ML) techniques, pure simulation techniques, and other techniques.

To begin with ML techniques, Fan et al. [5, 10, 11] aim to predict the hourly productivity of HTs (directly related to TT) in surface oil sand mines. They first apply a Gaussian mixture model (GMM) to operational data, partitioning it into three classes of low, medium, and high hourly productivity. Subsequent ML models then predict hourly productivity based on these latent classes. The results show significant performance improvements when adding a GMM upstream: the adjusted $R^2$ goes from 0.23 to 0.75 when combined with multiple linear regression (MLR) [11] and from 0.48 to 0.90 with Random Forest (RF) [10]. They thus conclude that using GMM before predictive models greatly enhances prediction quality.

Li et al. [8] also demonstrate the effectiveness of stacked learning for predicting the duration of HT transport cycles in an underground mine. They differentiate six sections of the route and use three predictive models (support vector machine (SVM), Light Gradient Boosting Machine (LightGBM), and RF) for these sections. An eXtreme Gradient Boosting (XGBoost) meta-model combines these predictions to estimate the total transport cycle duration. Their results show a mean absolute percentage error (MAPE) ranging from 2.31 to 4.56% across sections, outperforming other methods and implying the superiority of stacked learning.

We also note the work of Choudhury and Naik al. [12] which focuses on reducing operational costs in an open-pit mine by predicting HT TTs. They segment the transport cycle into distinct phases and use an RF algorithm, surpassing SVM and k-nearest neighbors (kNN) with a MAPE of 0.06%. RF is then used to optimize the HT allocation, demonstrating the effectiveness of ML for these predictions. Similarly, Sun, et al. [7] develop a method to predict HT TT by route section in open-pit mines using kNN, SVM, and RF models. They demonstrate that SVM and RF outperform kNN, with a substantial reduction in MAPE, particularly when weather data is incorporated into the models.

Other researchers proposed an integrated approach. For instance, Chanda and Gardiner [13] compare an artificial neural network (ANN), MLR, and simulation via TALPAC to predict truck cycle times. ML models are significantly more accurate than simulations, highlighting the superiority of ML methods in this prediction context. Similarly, Ristovski et al. [14] propose an ML, optimization, and simulation approach to predict TT in two open-pit mines. Their generalized linear model (GLM) with Gamma distribution outperforms benchmark models like random walk and historical TT average, showing better accuracy with a root mean square error (RMSE) of 49.7 s compared to 81.7 and 62 s for the benchmarks.

In addition, Ao et al. [15] use a long short-term memory (LSTM) neural network to predict TT in open-pit mines. LSTM achieves a mean absolute error (MAE) of 0.85 s and a MAPE of 2.6%, outperforming regression SVM and backpropagation neural network (BPNN).

Finally, Otunoye and Temeng [16] propose an MLR-based HT allocation model for open-pit mines, assisted by vehicle simulation. The MLR is trained to predict simulated TT, showing satisfactory TT prediction.

Other researchers used simulation techniques. For instance, Baek and Choi [17] simulate HT ore transport operations in an underground mine using beacon recognition data. Their discrete-event simulation model predicts TT based on statistical distributions of historical TT, demonstrating good accuracy. Upadhyay et al. [18] also explore the use of a discrete-event simulation model for an open-pit mine, combined with a mixed-integer linear programming (MILP) model. Their simulation model is validated by comparison with real data, confirming its applicability. In their second paper, Upadhyay et al. [19] present a Monte Carlo simulation model to estimate the hourly productivity of an HT fleet in open-pit mining operations. Their model is validated by comparison with the actual historical data of an oil sand mine, demonstrating superior accuracy.

Other techniques were also used in the literature. Erarslan [20] estimates truck cycle times using performance graphs and slowdown graphs for any road profile (i.e., speeds predicted by the manufacturer depending on the positive or negative slope encountered). He uses cubic spline interpolation to model the total resistance and corresponding HT speed, validating his approach through simulation. Finally, Gun et al. [21] use a MILP to schedule HT routes in an open-pit mine. Their approach significantly reduces the total TT of the HT fleet, especially when the number of active HTs is high.

The state-of-the-art in HT TT prediction research encompasses a wide variety of methodologies and models, as well as diverse prediction objectives. Notably, most papers focus on open-pit mines, with only two documents addressing underground mines [8, 17]. This underrepresentation may be due to inherent challenges in underground mining contexts, such as the lack of GPS, unstable transport routes, and complex road networks. Most papers utilize ML models, with only a few employing simulation methods. Chanda and Gardiner [13] combined both approaches, demonstrating that ML models can significantly outperform simulation software like TALPAC, which remains a popular tool nonetheless. The most effective ML models identified are generally XGBoost, RF, LSTM, SVM, gradient boosting regression (GBR), and Bayesian regularized neural network (BRNN). Using a GMM upstream and a stacking model downstream appears to be a relevant approach. Some methods predict average hourly productivity or a statistical distribution of it, while others predict cycle times, TT, or a statistical distribution of these durations. The best performances observed are generally shallow as (i) authors often use input variables that are unavailable for mining planners or difficult to exploit, limiting their applicability for short-term planning, (ii) data is often insufficient for predictive model training, (iii) the objective function is sometimes the mean TT or the total daily productivity and (iv) the performances were measured on all routes simultaneously. In the last case, the model uses input variables such as travel distance and elevation gain, which are naturally highly correlated with the upcoming TT. This leads to seemingly excellent results compared to benchmark models such as the average TT of HTs for all combined routes. These performances do not represent the model’s short-term predictive capabilities for a single given route, where precise prediction of upcoming work shift’s TT would be most valuable for planners. Indeed, the set of available explanatory variables would be far less correlated to TT. Some of the issues mentioned above are evident in the only paper focusing on underground mines using ML models, authored by Li et al. [8]. They used a small data sample of 200 travel instances gathered in 2 weeks (i.e., no seasonal effects), including real-time variables (unknown before the upcoming work shift) such as the number of times pedestrians were evaded, whether the ramp traffic light was red or not, and the oxygen concentration at the moment of travel. These limitations underscore the need for more robust methodologies that rely exclusively on pre-shift accessible variables, enabling better short-term planning—a gap this study aims to fill.

## Data Collection

Before presenting our proposed approach, this section describes the mining site under study, outlines the variables of interest, and details the data collection process.

### Case Description

**Fig. 1:**
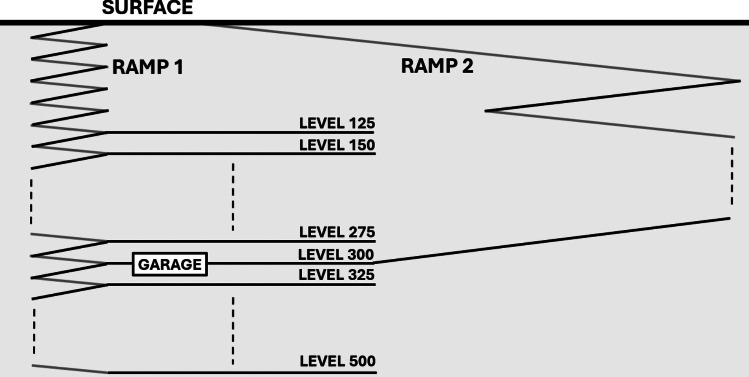
Fundamental architecture of Mine 1

The underground mine under consideration, referred to as “Mine 1,” utilizes the long-hole stoping method. As shown in Fig. 1, its layout features two ramps that provide surface access and service multiple operating levels. Ramp 1 serves all levels, from 125 to 500 m deep, with a 25-m interval between each level. Ramp 2 connects directly to Level 300, which provides access to Ramp 1 and the other levels. Level 300 is particularly important as it houses the main underground garage.

**Fig. 2:**
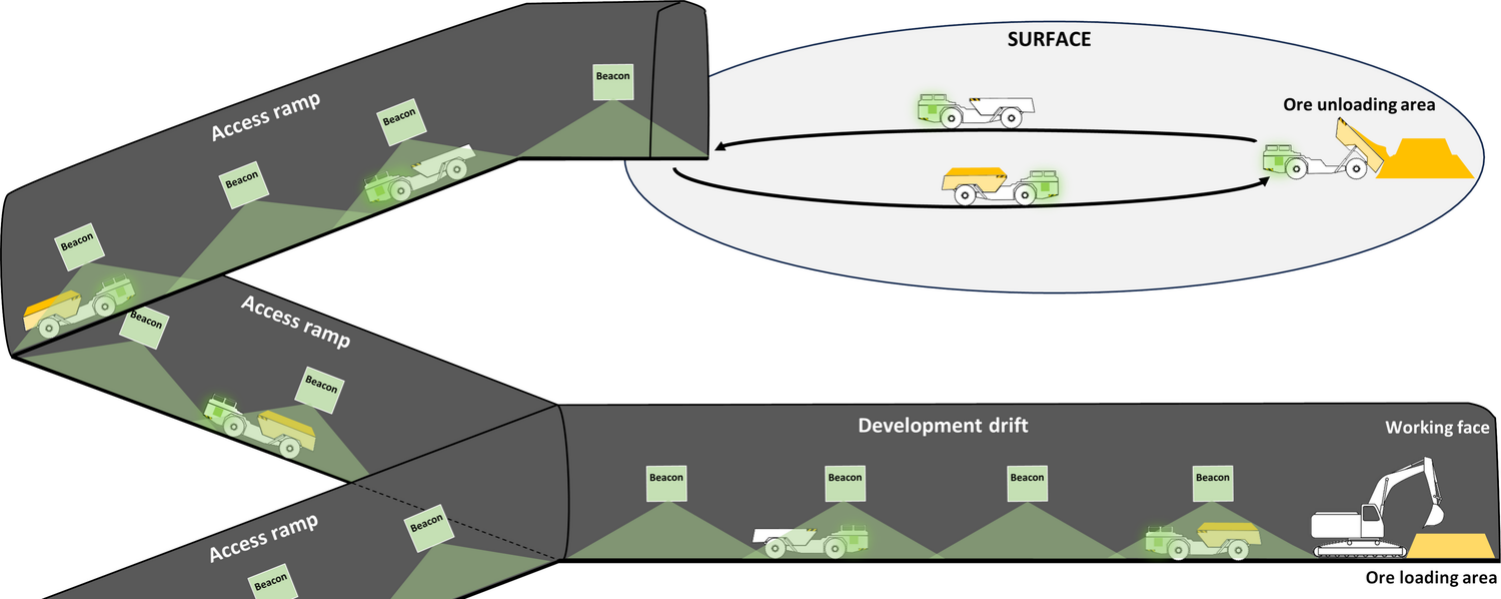
Overview of HT transport activities in Mine 1

Up to 11 HTs operate during two daily shifts: day and night. Refueling, breaks, and technical stops occasionally interrupt the hauling cycle.

As noted, various underground constraints (e.g., narrow ramps, complex traffic management, and limited visibility) strongly influence TTs. Mine 1 features parking chambers along the ramps to facilitate HT crossings, prioritizing loaded, ascending HTs. Since most extraction activities occur at deeper levels, HTs spend a significant amount of time navigating steep ramps, often encountering delays. Figure 2 provides an overview of transport activities at Mine 1. Green elements represent the underground connectivity network, including 135 vehicle localization beacons with their RFID detection zone, as well as the RFID chips mounted on each HT. Many of the beacons are located at key points, such as major intersections and the underground garage. Since the HTs travel partly on the surface, it is worth noting that Mine 1 experiences frigid temperatures, along with significant variations in humidity and precipitation throughout the year.

### Variables of Interest

In consultation with Mine 1’s operations supervisors, we identified several variables of interest influencing TTs:

- studied itinerary (start/end points, direction);
- HT type/model;
- HT identifier (accounting for individual characteristics);
- number of active HTs on the studied route;
- very long pauses (excluded as force majeure events);
- shift (day/night);
- day of the week;
- time of the year (for seasonal variations);
- temporal position within the work shift; and
- temporal position in the overall data timeframe (for identifying complex temporal relations).

Other relevant variables (e.g., driver identifier, HT mileage, HT load on ascending routes) were unavailable due to data limitations or confidentiality concerns.

Additionally, terrain factors and lane layout are not relevant inputs, as our prediction model is trained separately for each route. The historical TTs, therefore, implicitly capture fixed road-condition elements, such as lane layout and terrain factors.

**Fig. 3:**
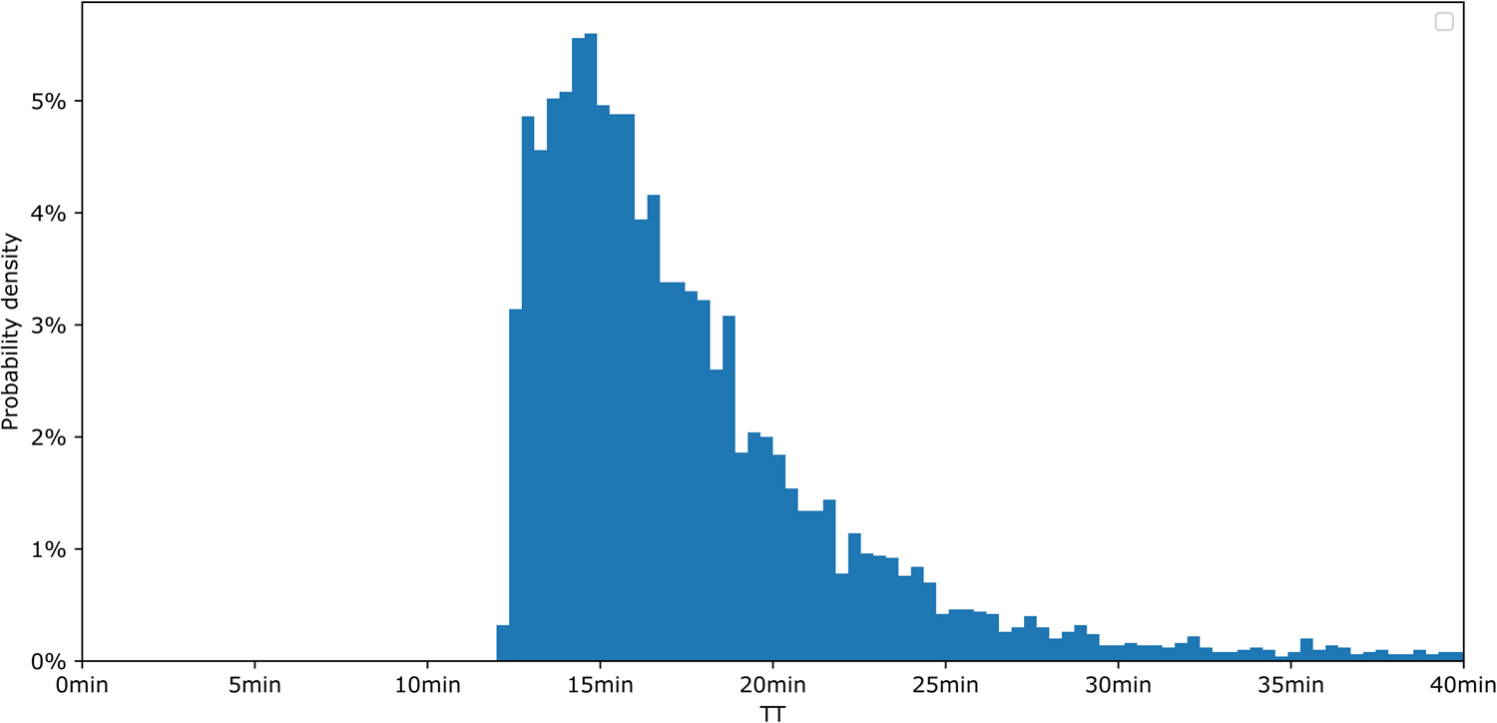
Histogram of observed TTs on the Surface$\rightarrow$Level350 route via Ramp 2

### Data Extraction

Beacon detection data, including the beacon identifier, HT identifier, and timestamp, can be used to infer each variable of interest. By analyzing detection sequences, HT trips can be reconstructed, and contextual variables can be derived.

More than three million records of HT detection data were extracted over a nearly 3-year period. The mine’s plan, which indicates beacon positions, helped interpret these sequences and understand local conditions.

### Data Preparation

Initially, we utilize the depth data associated with each beacon to verify the consistency of vertical HT movements and evaluate the consistency of route directions. We rectify any inconsistencies and outdated identifiers. Checkpoint ramp beacons at each level divide itineraries into shorter sections.

The beacon system at Mine 1 requires careful handling during data preparation. In principle, a beacon registers a timestamp when an HT enters its detection zone, and only if the previous detection for that HT came from another beacon. However, in Mine 1, the detection cones are very wide; HTs can trigger detections at distances exceeding 100 m if no obstacle blocks the signal, resulting in confusing localization data. Furthermore, the heavy overlap between detection zones frequently leads to conflicts, where two adjacent beacons each consider they have newly detected the same HT, generating sequences of redundant detections. Beacon detection attempts occur every two seconds. Due to these factors, raw detections must be cross-checked with preceding and following detections to reconstruct plausible trajectories and eliminate ambiguities.

Trips containing very long pauses (lasting over 30 min) were excluded. We developed a trip recognition algorithm to identify trips between pairs of beacons and compute TTs. We iteratively refined this algorithm to handle detours, remove rare trips detected between shifts, and resolve other inconsistencies.

The descending route from the surface to the entry of Level 350 via Ramp 2 (“Surface$\rightarrow$Level350”) was selected as a key ore transport route. This route passes through Level 300 (with the garage), involves significant elevation changes, and yields over 5000 complete trip observations. Figure 3 shows a histogram of the resulting TTs, which are highly skewed and vary more than threefold.

**Fig. 4:**
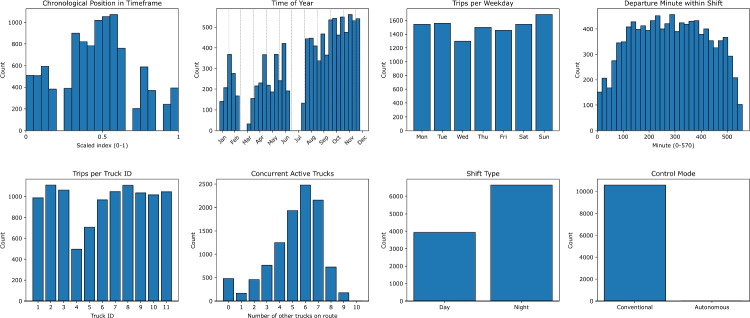
Historical distributions of the input variables on the Surface$\rightarrow$Level350 route

The remaining variables were then extracted. The number of active HTs per shift on the studied route was determined by counting detections at the route’s beacons and applying a threshold of 50 detections per shift. Work shifts, days of the week, and seasonal variations were derived from timestamps. The within-shift time and the overall timeframe position were also computed. Figure 4 summarizes the empirical distribution of each predictor.

**Table 1:** Predictor variables: type and encoding

Variable	Type	Encoding
Chronological position in timeframe	Continuous	Min–max normalization (−1$\rightarrow$1)
Time of year	Continuous cyclic	Sine/cosine transform
Weekday	Discrete cyclic	Sine/cosine transform
Departure minute within shift	Continuous cyclic	Sine/cosine transform
Truck ID	Categorical	One–hot encoding
Concurrent active trucks	Discrete	Min–max normalization (−1$\rightarrow$1)
Shift type (day/night)	Binary	0 = day, 1 = night
Control mode (conventional/autonomous)	Binary	0 = conventional, 1 = autonomous

### Data Preprocessing

The following preprocessing steps were applied:

- HT identifier: One-hot encoding (see [22])
- Number of active HTs: Normalized to [−1, 1]
- Work shift: Binary (0/1)
- Date of trip (relative timeframe): Normalized to [−1, 1] and scaled by −1 to reflect potential gradual TT decreases over time on a given route due to efficiency gains
- Cyclic variables (day of the week, day of the year, within-shift time): Represented with sine/cosine transforms to preserve cyclicity and aligned seasonally with expected conditions

For ascending trips, TTs are normalized directly since the skewness is minimal. For descending trips, a logarithmic transformation is applied to reduce skewness. After that, some outliers are eliminated, and the distribution is normalized. TT predictions will be denormalized and exponentiated as necessary.

The type of each predictor and the corresponding encoding strategy are summarized in Table 1.

After preprocessing, the variables are ready for use by the ML models evaluated in the next section. We denote the predictors as *X* and the transformed TTs as *y*. The dataset is split into training ($X_{\text {train}}, y_{\text {train}}$) and test sets ($X_{\text {test}}, y_{\text {test}}$) with a 70:30 ratio.

## Methods

Previous work reviewed in Section 2 shows that generic statistical techniques and Monte Carlo or discrete-event simulations, TALPAC included, are consistently outperformed by ML models on the trip-level TT prediction task. While these traditional methods can reproduce an overall distribution of HT TTs, they do not provide per-trip predictions conditioned on variables that change from one run to the next, such as weekday, shift, truck identifier, and expected traffic. ML models, by contrast, explicitly ingest these operational variables and achieve markedly lower errors.

**Fig. 5:**
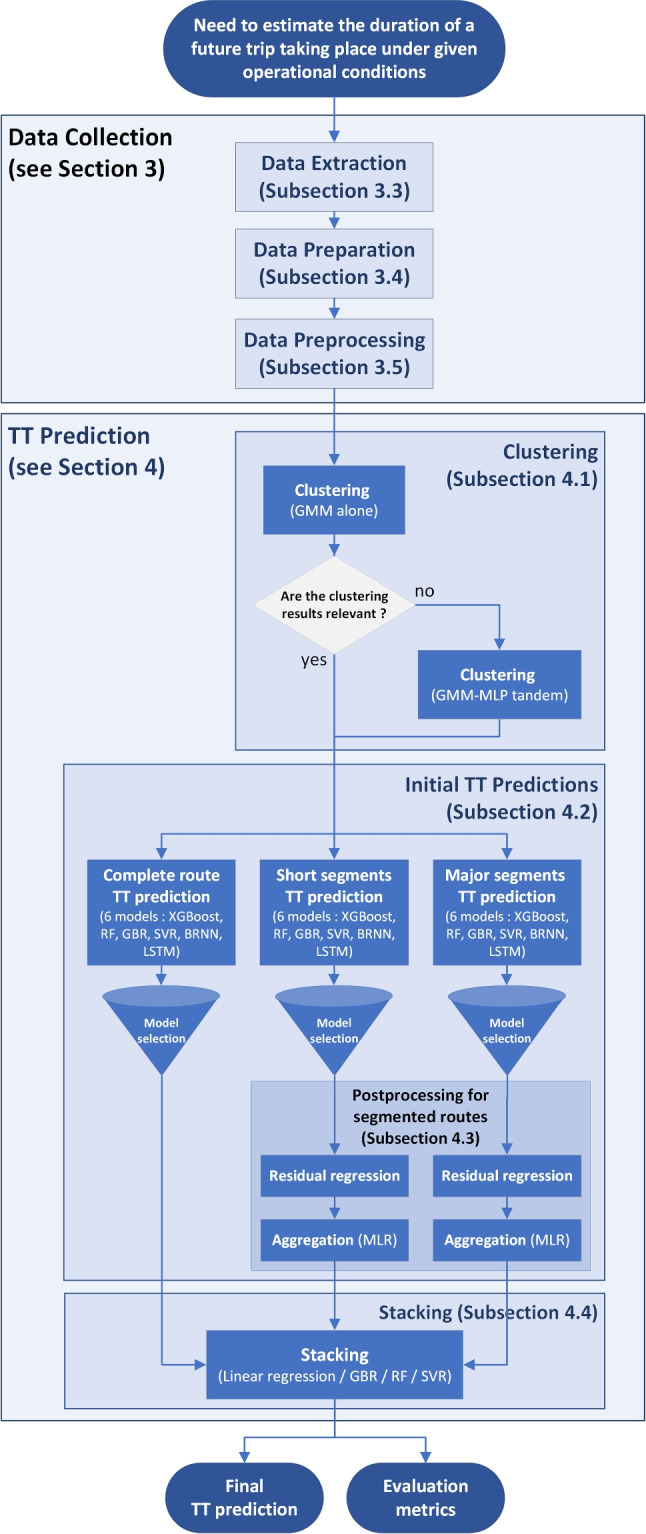
Flowchart of our methodology

Against this background, we developed a route-specific ML workflow that aims to enhance both accuracy and interpretability. It proceeds in four main steps: (i) clustering, (ii) initial TT prediction with several models, (iii) post-processing for segmented routes, and (iv) a stacking model that aggregates the various predictions. Figure 5 provides an overview of the proposed method.

### Clustering

As discussed in Section 2, Fan et al. [5, 10, 11] demonstrated the effectiveness of using GMMs to cluster HT travel data in surface mining. GMMs can approximate complex, multimodal distributions by combining several Gaussian components [23]. Instead of summarizing the TT distribution with a single statistic, GMMs thus allow each observation to be associated with one of several latent “speed regimes” (e.g., very fast, fast, nominal, slow, very slow). In our study, GMMs are applied to the TT training data to identify these latent regimes, which are then appended to the feature vector used by the downstream prediction models.

However, unlike the studies by Fan et al., our context involves significant differences. These surface mine studies predicted TT for all routes simultaneously, with clear patterns driven by factors such as travel distance and empty HT speed. In contrast, our GMM must capture subtler latent relationships, given the underground mining context and route-specific predictions. The use of a GMM alone could thus be potentially insufficient. If necessary, we propose enhancing the GMM clustering with a Multi-Layer Perceptron (MLP). MLP is a neural network composed of layers of interconnected nodes, capable of modeling complex relationships and suitable for classification and regression tasks [24]. In our GMM-MLP tandem approach, the GMM fits the distribution of observed TTs and partitions them into distinct clusters. Then, the MLP uses operational conditions to predict the probability that a future travel belongs to each cluster. These cluster membership probabilities are appended to the original feature vector to further enhance the TT prediction.

### Initial TT Predictions

We predict TTs at three complementary levels of route segmentation, because each level presents a distinct trade-off.

- **Complete-route prediction:** predicting the total TT in a single step is straightforward and minimizes error propagation. Still, it can mask local variability caused by intersections or changes in operational conditions.
- **Short-segment prediction:** dividing the route into short inter-level segments offers two advantages: (i) Far more observations per segment than per complete route, because many itineraries share the same small sections; and (ii) The ability to tailor predictions to very specific road contexts. Its drawbacks are that the final TT must be reconstructed by summing segment predictions, allowing minor localization errors to accumulate, and the model is trained on trips that are not always part of the entire target route, introducing bias. For example, a HT may slow down at the end of the segment to turn, versus maintaining speed to continue straight onto the next segment.
- **Major-segment prediction:** using long, well-defined ramp segments or drift sections provides intermediate granularity, fewer summations than the short-segment approach, yet more detail than the complete-route approach.

Because the significance of each granularity varies with the mine and route, we retain all three to feed the final stacking model.

We evaluate six ML models identified as top performers in the literature: RF, XGBoost, GBR, SVM, BRNN, and LSTM.

- **RF:** An ensemble of decision trees that averages multiple predictors, improving generalization and reducing overfitting [25].
- **XGBoost:** A gradient-boosted tree method known for its efficiency, regularization, and high performance [26].
- **GBR:** A model that builds ensembles of trees stage-wise, each successive tree correcting the residuals of the previous ones [27].
- **SVM:** A flexible model that, when adapted for regression, uses kernel functions to fit complex patterns, finding an optimal hyperplane in feature space [28].
- **BRNN:** A model based on Bayesian principles, which outputs probability distributions and provides robustness in noisy environments [29]. For deterministic predictions, we use a regularized neural network inspired by BRNNs, incorporating L2 regularization to constrain weights and dropout to manage uncertainty. This approach approximates the central values of the distributions produced by a BRNN.
- **LSTM:** A recurrent neural network architecture designed to handle long-term dependencies [30]. LSTMs are well-suited for time-series modeling and leverage historical context (e.g., recent trips on the same route) to capture short-term operational dynamics.

### Specific Postprocessing for Segmented Routes

We apply a two-step postprocessing approach to address the two segment-prediction biases identified in Section 4.2.

First, we apply residual linear regression to adjust segment-level predictions. This step reduces discrepancies between predicted segment TTs and those observed exclusively during complete trips. Residual regression enhances accuracy by refining predictions based on residual patterns from the initial model, as outlined in [31] and [32].

Second, we aggregate corrected segment predictions into a final TT estimate. An MLR model ensures that the aggregation aligns with observed complete-route TTs. The regression equation is:

$$ TT_{complete}\left( TT_1, TT_2, \dots , TT_n\right) = \sum _{i=1}^n k_i TT_i + C, $$

where $k_1, k_2, \dots , k_n$ are positive coefficients and $C$ is a constant. A custom cost function combines the mean squared error (MSE) between predicted and observed complete TTs with a regularization term:

$$ \text {Cost} = \text {MSE} + \lambda _{\text {reg}} \sum _{i=1}^n (k_i - 1)^2. $$

The regularization term penalizes deviations of $k_i$ from 1, ensuring realistic aggregation. The parameter $\lambda _{\text {reg}}$ is manually tuned to strike a balance between accuracy and stability.

This process corrects additional biases and produces more consistent, accurate, and complete-route TT predictions. The combination of residual regression and robust aggregation minimizes errors while maintaining practical interpretability.

### Stacking

We combine predictions from the various segmentation strategies using a stacking model. Stacking enables the final model to leverage the strengths of each segmentation technique, thereby enhancing precision and robustness. Several models (e.g., linear regression, RF, GBR, and SVM) are tested as stackers.

### Hyperparameter Optimization

Hyperparameter optimization is conducted to ensure that each model operates under its best-performing configuration [33]. For the GMM, the optimal number of components is selected using the AIC, ensuring a suitable balance between complexity and predictive performance [34, 35].

For each model, systematic or randomized search procedures are applied over large hyperparameter ranges. Specifically, the stacking model is jointly optimized via the optuna library [36] to select the best-performing model among those tested and fine-tune its hyperparameters.

A comprehensive list of the considered hyperparameters and their respective search ranges is provided in Table 6.

**Fig. 6:**
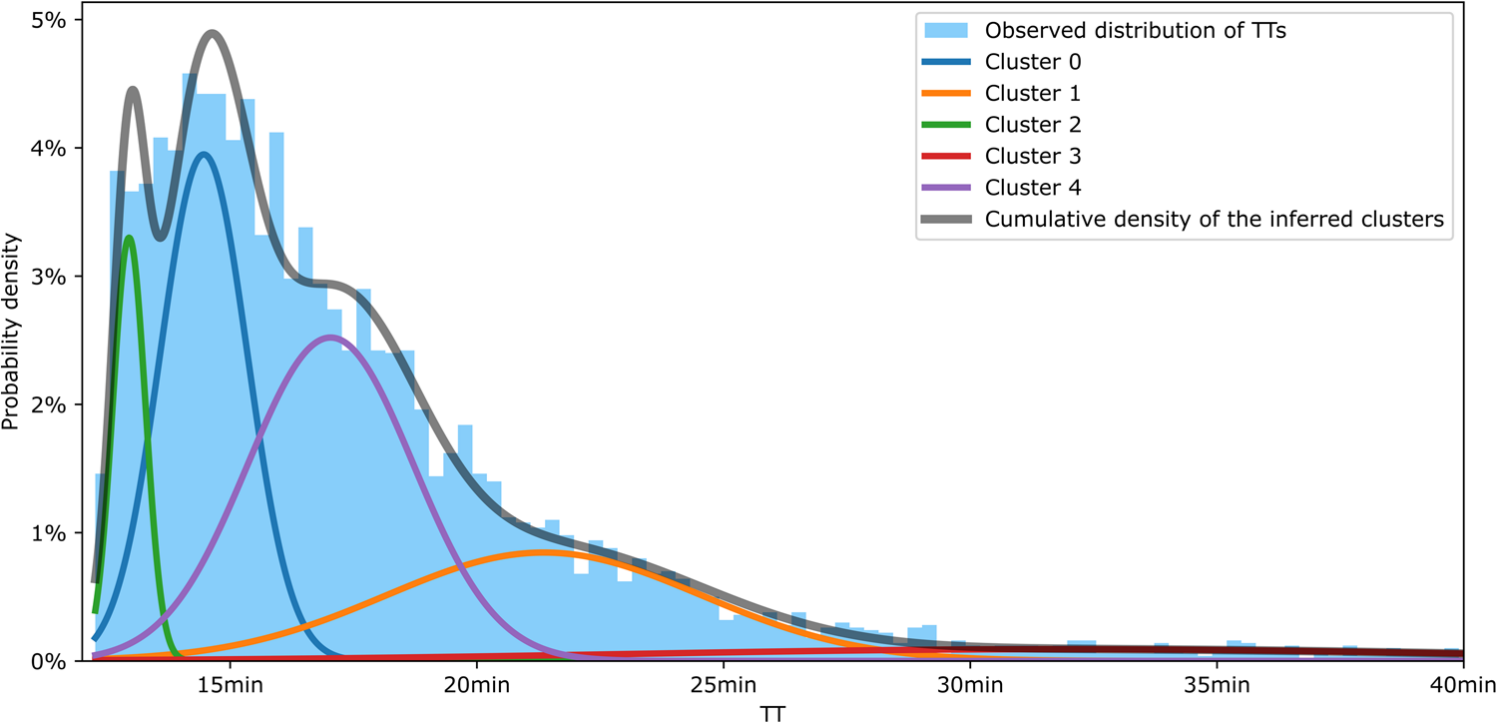
Inference of five clusters (Gaussian components) by the GMM in $y_{train}$

**Fig. 7:**
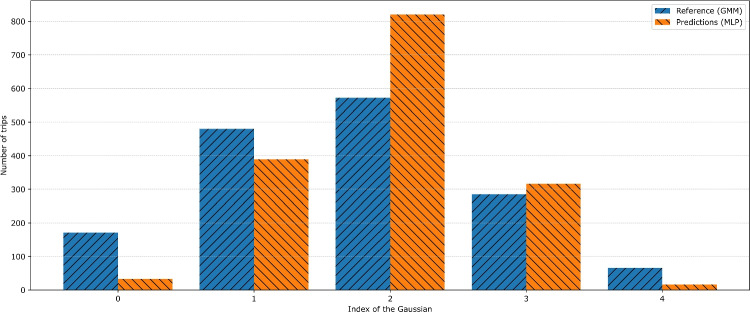
Bar chart of the most probable group, as determined by the GMM (reference) and predicted by the MLP

### Evaluation Metrics

We adopt multiple-fold cross-validation to mitigate biases and improve the reliability of performance estimates. To capture variations and outliers, we rely primarily on root mean square error (RMSE):

$$ RMSE = \sqrt{\frac{1}{N} \sum _{i=1}^{N} (y_i - \hat{y_i})^2}. $$

Additionally, mean absolute error (MAE) is reported to provide a complementary perspective:

$$ MAE = \frac{1}{N} \sum _{i=1}^{N} |y_i - \hat{y_i}|. $$

We benchmark model performance to the historical TT average for the studied route, a widely used reference in the mining industry [14].

## Results

This section presents the results of the integrated model for predicting TTs on the selected route in Mine 1 based on the previously explained theoretical framework.

### Adopted Segmentation

Following our methodology (Fig. 5), we trained our model to predict TTs for the entire selected route (Surface$\rightarrow$Level350) as well as for two alternative segmentations. The first segmentation divides the route into short segments of 25-m elevation changes. The second identifies a major segment (Surface$\rightarrow$Level300, a simple ramp without level access) and then splits the remaining portion into short segments (see Fig. 1).

### Results and Evaluation

In this subsection, the results of each sub-model are presented in the order they appear within the overall prediction model. Hyperparameter optimization was performed at each stage, with optimal parameters listed in Table 6 for the different segmentation types.

The GMM alone failed to identify relevant groups in $X_{train}$, as indicated by the continuously decreasing AIC without convergence. It suggests that the input features are significantly less directly correlated with TTs than in Fan et al.’s prior work [5, 10, 11]. Consequently, we adopted the GMM-MLP approach described in Section 4.1. We first applied a GMM to $y_{train}$, and the AIC suggested that five clusters were optimal. These clusters collectively aligned closely with the TT distribution, as shown in Fig. 6, where the five inferred Gaussian densities are represented in color, and the grey curve depicts their cumulative density. After training the MLP, it produced relatively accurate cluster membership probabilities for $X_{test}$, as illustrated in Fig. 7. Although extreme classes were assigned the highest probability less frequently, the resulting estimates remained coherent with the underlying distribution.

Once hyperparameters for each ML model are optimized, we apply them to $X_{test}$. The LSTM and BRNN achieved the highest accuracy for complete-route predictions (Table 2), although the BRNN only produced predictions that approach the historical average. For short-segment route predictions, the LSTM and XGBoost demonstrated superior performance. For major-segment predictions, the LSTM reduced RMSE and MAE by 10% relative to other models. On the major segment Surface$\rightarrow$Level300 specifically, the LSTM outperformed the benchmark and all other models by 20% regarding the RMSE.

Based on these results, we selected the LSTM for initial TT predictions. Incorporating GMM-MLP cluster predictions as input, instead of using no clustering, provided slight improvements, reducing RMSE by 3% and MAE by 5%.

**Table 2:** Performances of six ML models and the benchmark for the complete route

Model	MAE	RMSE
Benchmark (average TT)	213	307
BRNN	204	307
XGBoost	214	312
RF	210	312
GBR	208	309
SVM	226	335
LSTM	199	312

Applying residual regression on segmented routes further improved results. For short segments, RMSE dropped by 2.5%, and MAE by 1%. For the major-segment approach, RMSE fell by 13%, and MAE by 15%. Introducing aggregation via MLR after residual regression improved RMSE by 6% for short segments and by 2.5% for the major-segment approach, though MAE slightly worsened.

Table 3 compares final performances after applying residual regression and aggregation. Predictions based on the major-segment approach improved by 10% in both RMSE and MAE compared to the complete route.

Our final stacking model further enhanced accuracy, reducing RMSE by 9% and MAE by 12%.

Ultimately, when comparing the benchmark’s predictions to our final model, we observed a significant cumulative performance gain, with an 18% reduction in RMSE and almost a 29% reduction in MAE.

**Fig. 8:**
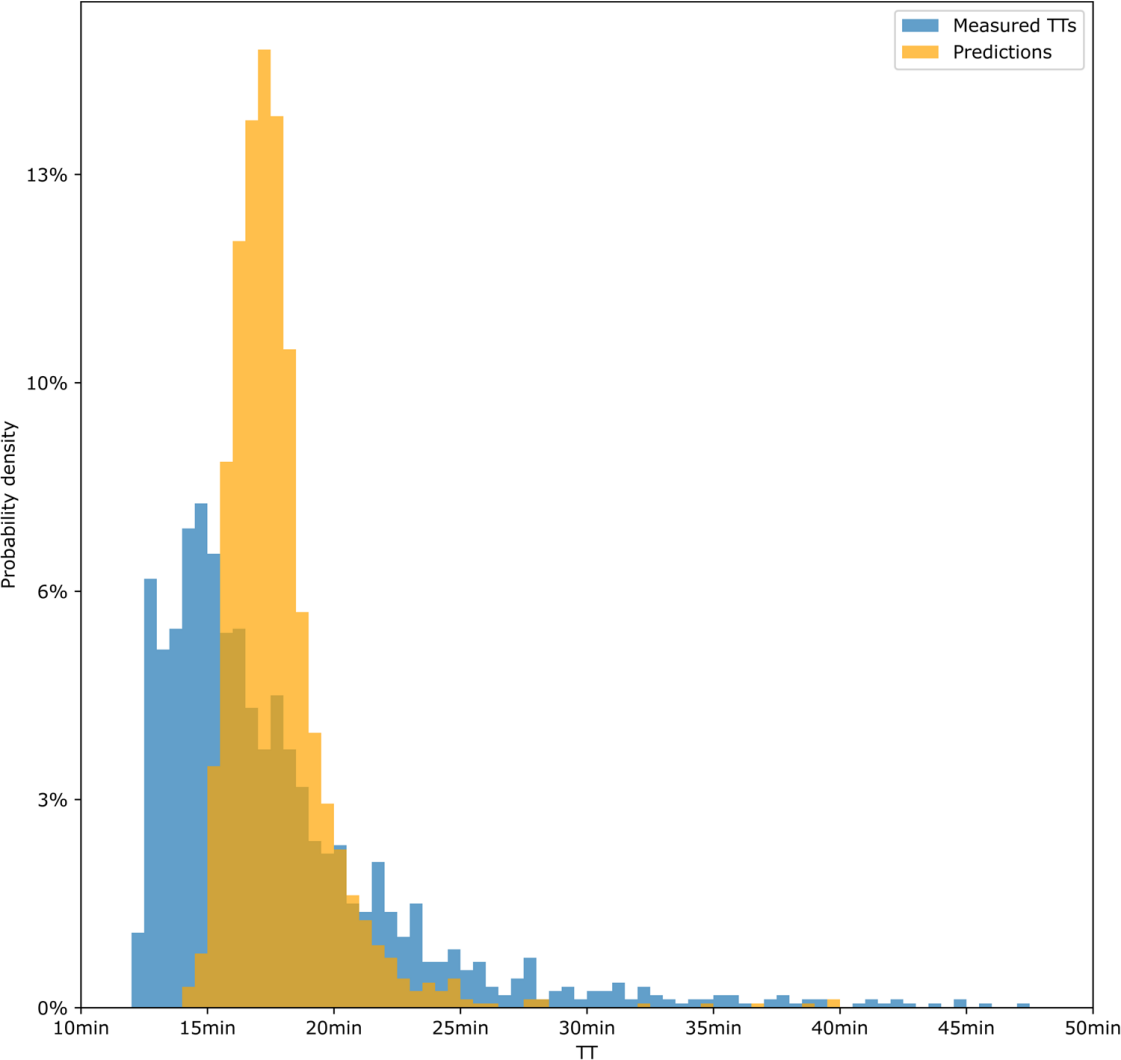
Histograms of measured and predicted TTs by our stacking model

Figure 8 compares the distributions of measured TTs and stacking model TT predictions. Although the stacking model is more accurate, the predicted distribution differs from the historical one, showing a secondary mode with a lower amplitude.

**Table 3:** Final performances of the benchmark, initial prediction models, and the stacking model

Model	Segmentation	MAE	RMSE
Benchmark	Complete	**213**	**307**
LSTM	Complete	199	312
	Short	208	322
	Major	179	278
Stacking (GBR)	All three	**158**	**252**

Boldface values represent the reference or final results for each technique

Predicting TTs on the inverted ascending route (Level350 $\rightarrow$Surface) proved easier, reducing RMSE by 34% compared to the benchmark. This corresponds to a 20% lower RMSE than on the original descending route, proportionally. This result underscores the difficulty of predicting TTs on a **descending** route, where trucks must yield to those moving uphill.

As an experiment, we raised the frequency of the feature that logs HT activity levels on the monitored route from once per shift to every thirty minutes. This adjustment significantly boosted model accuracy, reducing the LSTM’s RMSE by 9% and MAE by 20%. Consequently, precisely forecasting these activity levels prior to each shift could substantially improve TT predictions. To remain conservative, we do not account for these potential improvements in our final results, as such forecasting may not always be feasible prior to shifts.

### Synthesis of Results for Mine 1

The integrated model surpassed the industry benchmark for a key route in Mine 1, regardless of direction, despite anomalously long trips. The model’s composition—GMM-MLP for clustering, three segmentation approaches (all processed by LSTM), residual regression and aggregation for segmented routes, and final stacking—was fixed following extensive testing, confirming the effectiveness of this multi-step approach.

## Generalization Test

This section evaluates the model’s generalization capability by applying it to a second mining site called Mine 2.

### Study Case Description

Mine 2 features a simpler architecture, with a single ramp serving all levels. The shallowest level is located at a depth of 30 m, and the deepest at 450 m, with 30-m inter-level gaps. A washing bay is located at a depth of 60 m. Seven HTs operate simultaneously, including two fully autonomous weekly shifts. During these shifts, an autonomous driving system resolves route conflicts using chambers dug into the ramp’s walls. The vehicle detection system comprises over 200 beacons, differing in design and configuration from Mine 1. Lastly, Mine 2 is located in a more temperate region.

**Fig. 9:**
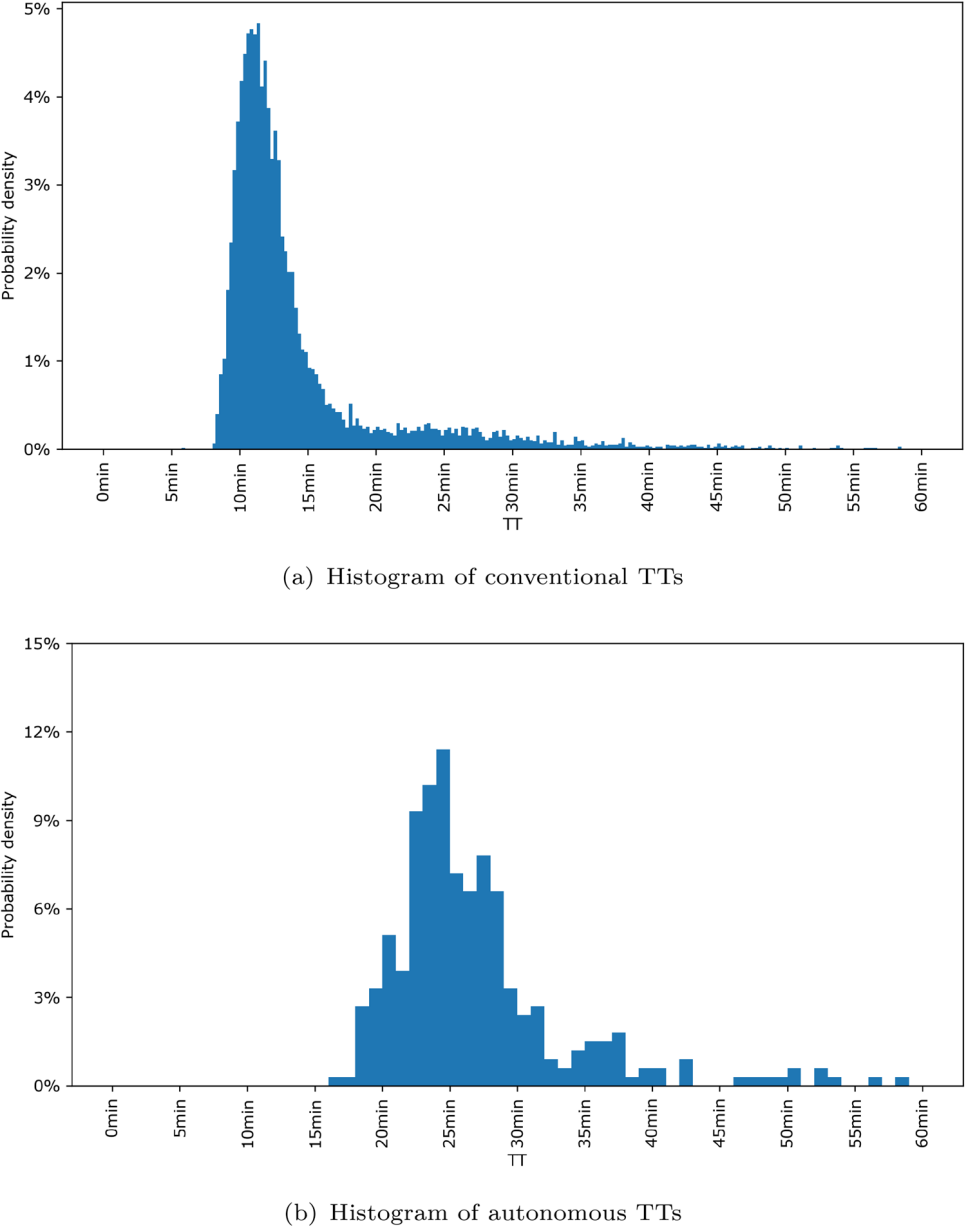
Histograms of TTs by driving mode for Level30$\rightarrow$Level300 in Mine 2

### Data Preparation

The variables of interest are the same as in Section 3.2. This time, the “Type of HT” feature is crucial for distinguishing between autonomous and conventional trips.

Over 2 years of data were collected.

At Mine 2, the beacon system is configured with narrower detection cones (approximately 5–10 m radius) than in Mine 1 and attempts to detect HTs every second. In this configuration, detections are generally more localized and reliable: an HT triggers a detection only when it is genuinely close to the beacon, and conflicts between adjacent beacons are rare.

However, beacon malfunctions were significant. Entire blocks of beacons stopped functioning, sometimes for months, making route recognition challenging. To mitigate this, we made our route recognition algorithm more flexible, which should increase the number of non-standard trips recorded. We also revised checkpoint selection to maximize functional overlap between beacons.

Based on an analysis of predominant routes, we select the Level30$\rightarrow$Level300 route. A total of 7742 conventional trips and 321 autonomous trips are identified. Because of beacon malfunctions, only 2.66% of conventional trips had detections from all checkpoints. Figures 9a and b show the TT distributions for each driving mode on the Level30$\rightarrow$Level300 route. Autonomous trips generally take twice as long due to conservative safety protocols.

Despite the beacon malfunctions, the right tail of the conventional TT distribution is significantly thinner than that of Mine 1. This observation suggests that the number of non-standard trips detected is likely to be low. The beacon installation strategy, with more evenly distributed beacons and a significantly narrower detection field compared to Mine 1, appears to be the most critical factor for accurately recognizing trips and associated TTs. This strategy was much more refined and precise in Mine 2 compared to Mine 1. Moreover, it appears to outweigh the importance of the beacons’ reliability and their ability to consistently save detections. This finding is particularly unsettling, given the significant adjustments made to our route recognition algorithm.

The remainder of the data preparation process mirrors that of Mine 1, with adjustments to account for detection inconsistencies.

**Fig. 10:**
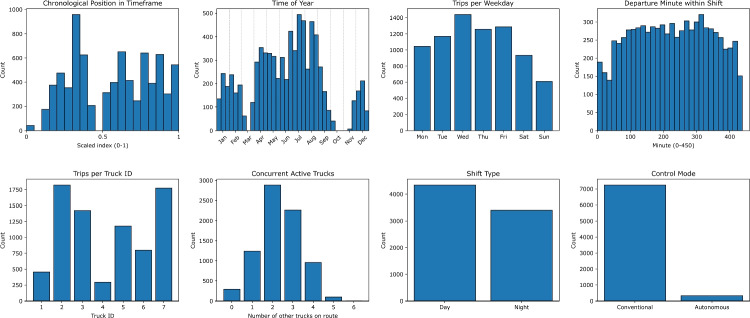
Historical distributions of the input variables on the Level30$\rightarrow$Level300 route

Figure 10 summarizes the empirical distribution of each predictor.

**Fig. 11:**
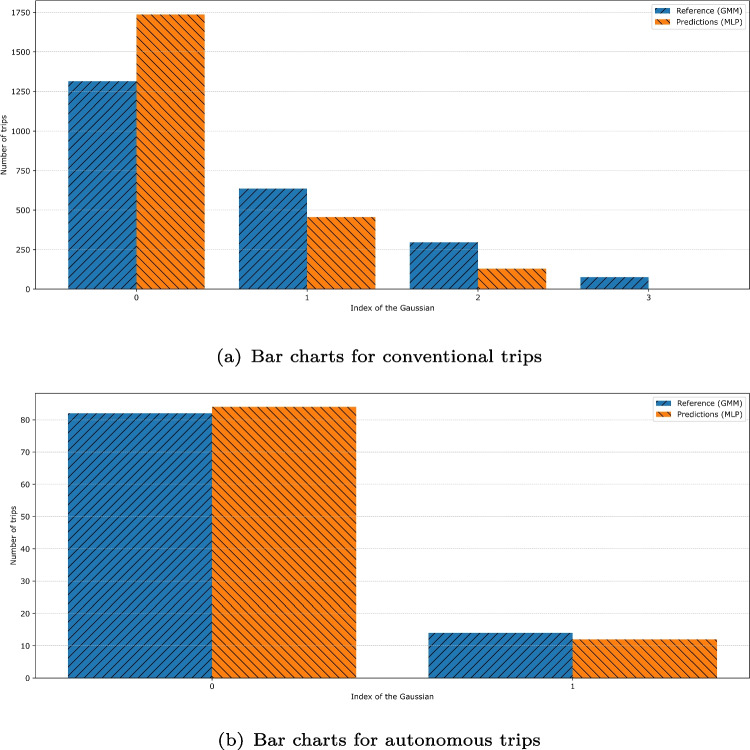
Bar charts of the most probable group, as determined by the GMM (reference) and predicted by the MLP, by driving mode for Level30$\rightarrow$Level300 in Mine 2

### TT Prediction

This subsection highlights TT prediction steps, with Table 6 compiling the optimal hyperparameter values.

In the major-segment approach, since the most reliable intermediary beacon is located at Level 120, we split the route into two segments: Level30$\rightarrow$Level120 and Level120$\rightarrow$Level300.

The clustering process remained consistent with Mine 1. The bar charts comparing the MLP’s cluster predictions to the clusters that the GMM would have assigned are shown in Fig. 11a and b for each driving mode. The results observed for autonomous trips are remarkably consistent, suggesting that the MLP effectively captures the relationship between TT and operational data in this context.

**Table 4:** Final performances of the benchmark model, initial prediction models, and stacking model

Model	Segmentation	Phase	Conventional		Autonomous	
			RMSE	MAE	RMSE	MAE
Benchmark	Complete	—	**392**	**254**	**435**	**300**
Init. pred.	Complete	—	**351**	**203**	**329**	**225**
	Short	LSTM	381	213	—	—
		Res. reg.	388	224	—	—
		**Final**	**373**	**248**	—	—
	Major	LSTM	378	218	—	—
		Res. reg.	356	204	—	—
		**Final**	**352**	**231**	—	—
Stacking	All three	—	**349**	**223**	—	—

Boldface values represent the reference or final results for each technique

Regarding the initial TT predictions, we only predict autonomous TTs for complete trips, as there is insufficient data. Beacon malfunctions challenged the use of LSTM networks: we imputed the missing historical TT values using a rolling window approach, allowing the model to continue making reasonably accurate predictions despite incomplete data.

We applied residual regression and aggregation without any additional adjustments, while identifying linear regression as the best stacking model.

### Results

Evaluation metrics are presented in Table 4.

The complete-route TT predictions achieved a relative reduction in RMSE of 10.5% and MAE of 20% compared to the benchmark, outperforming those in Mine 1. The prediction of autonomous TTs showed promising results despite a minimal sample size, achieving a mean relative reduction in RMSE of 24.5% and in MAE of 25%.

Segmented route predictions highlighted limitations. Regardless of the segmentation method (short or major segments), the LSTM alone achieves a slight reduction of the RMSE compared to predictions on the complete route (2.8% and 3.6%, respectively) and a significant relative reduction in MAE (16.1% and 14.2%, respectively). Applying residual regression to the results reveals an intriguing phenomenon: performance quality increases significantly with segmentation into major segments but worsens with short segmentation. For major segments, compared to the LSTM alone, we observe a relative reduction in RMSE of 5.8% and in MAE of 6.4%. For short segments, RMSE shows a relative increase of 1.8%, while MAE increases by 5.2%. We hypothesize that some short segments are underrepresented in complete trips due to beacon malfunctions, which hinders the residual regression model’s ability to generalize effectively. We thus recommend verifying that residual regression improves prediction results for each segment and avoiding applying it when it does not. Meanwhile, the aggregation tends to improve the RMSE and worsen the MAE, with a more significant loss in MAE precision than observed in Mine 1. Depending on the type of segmentation, the RMSE decreases by 3.9% and 1.1%, respectively, while the MAE increases by 10.7% and 13.2%. Despite this, we consider these results acceptable, as our models aim to minimize RMSE.

Finally, the stacking model achieves a lower RMSE than each of the initial TT prediction models and remains unaffected by the poor performance of the short-segment approach. However, the relative RMSE reduction is minimal (0.6% compared to the LSTM alone on the complete route), while the MAE increases significantly (9.9%).

Despite several challenges, the model ultimately achieved an 11% RMSE reduction for conventional trips and a 24.5% RMSE reduction for autonomous trips, validating its effectiveness in diverse scenarios.

## Conclusion

This article presents a significant advancement in the field of HT TT prediction in underground mines. It leverages detection beacon data solely—a novel approach in this field—while utilizing only inputs readily available to mining planners prior to each shift. The proposed methodology includes a robust strategy for ordinary trip recognition and feature engineering to extract variables available before each work shift, fully leveraging detection beacon data. Central to our approach is a triple segmentation strategy combined with a stacking model, which plays a decisive role in achieving both satisfying precision and overall robustness of the predictions.

Our methodology was tested on two distinct mining sites, demonstrating its adaptability and superior performance compared to the traditional industry benchmark, even in the face of operational variability and significant data quality issues. Notably, the proposed workflow is an architecture, not a fixed model. For any route in any mine equipped with a beacon system, the procedure consists of (i) preparing a training set from that route’s beacon detections and variables, then (ii) re-initializing and re-training the ML models, which takes only a few minutes on standard hardware. This adaptability was demonstrated on Mine 2, despite significant differences from Mine 1, including the layout of beacons, ramp geometry, and the use of autonomous trucks.

Our results showed that LSTM networks were particularly effective at capturing the temporal dependencies of HT underground operations, outperforming the other models that had previously stood out in the literature for HT TT prediction. Although the GMM-MLP tandem clustering showed some utility compared to GMM-alone clustering, its contribution was less significant.

Despite our model demonstrating robustness and performance, some limitations remain. The low quality of beacon detection data and specific constraints at each mining site can introduce significant challenges that are sometimes unsolvable during the data preparation phase. Additionally, we could not filter non-ordinary trips effectively without sacrificing a substantial portion of ordinary trips, which hinders the model’s accuracy. Missing checkpoint beacon data also necessitates costly computational adjustments, significantly degrading the accuracy of predictions. Notably, beacon coverage remains a prerequisite; underground mines lacking a beacon system must first install one. However, such systems are becoming increasingly commonplace.

Several avenues for improvement have been identified to overcome these limitations and further improve our methodology. First, enhancing beacon systems and monitoring the health of detection data through systematic beacon checks would ensure high-quality data collection and help prevent massive data losses. Second, better identification and exclusion of non-ordinary trips could be achieved by integrating additional attributes such as HT fuel level, engine speed, and dump bed lift status into the data preparation process, along with TT filtering thresholds set by HT activity experts for each segment and route. It would significantly enhance prediction accuracy when beacon data is insufficient to identify specific non-ordinary trips. Third, introducing new short-term prediction variables, such as the driver’s identifier, HT mileage, HT load (on ascending routes), or predictions of mine zone activity levels every half hour, would further boost model performance. Finally, adapting the model for real-time predictions by integrating real-time telemetry data as additional inputs could enable the model to be utilized in real-time management systems, using beacon detection data to predict HT TTs dynamically, for the first time.

Several avenues for improvement have been identified to overcome remaining limitations and further strengthen our methodology. First, although we focused on the most frequently traveled routes to maximize data availability and relevance, our framework can also be applied to less frequently traveled or newly planned routes. To this end, we could first predict the central, heavily trafficked section using the proposed ML model and then append reliable estimates (either geometry-based ML predictions or segment-specific historical means) for the remaining sections, thereby maintaining superior accuracy over simple historical averages. Second, enhancing beacon systems and monitoring their health through systematic checks will ensure robust data collection and prevent significant data losses. Third, improving the identification and filtering of non-ordinary trips by integrating additional attributes (e.g., fuel level, engine speed, dump bed status) alongside expert-defined thresholds will enhance prediction quality when beacon coverage is imperfect. Fourth, introducing new short-term predictors—such as driver identifiers, HT mileage, HT load on ascending trips, or forecasted activity levels at half-hour intervals—promises further performance gains. Finally, adapting the model for real-time deployment by integrating continuous telemetry with beacon data could enable dynamic, trip-level TT predictions within a real-time digital twin traffic simulator, opening the door to fully responsive fleet management.

## Figures and Tables

**Fig. 12:**
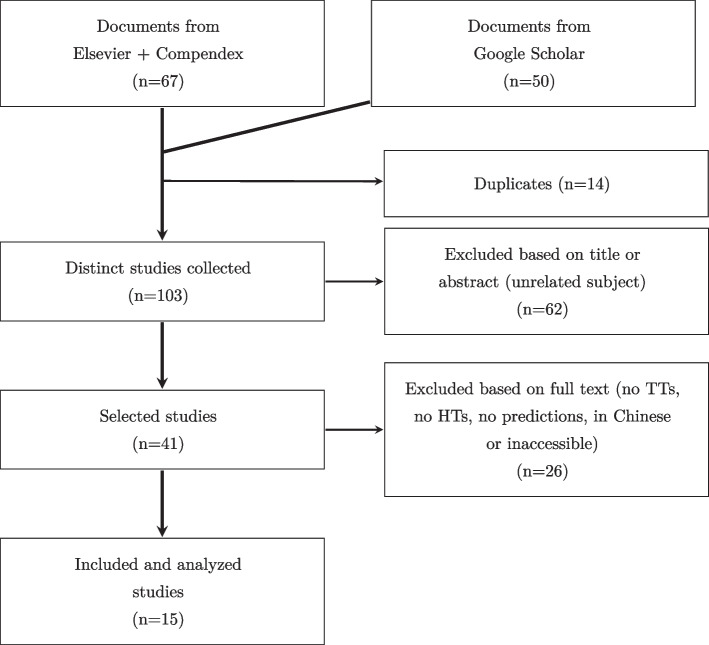
Article identification process

## Data Availability

The data used is confidential.

## Appendix 1: Systematic literature review

We conducted a systematic literature review addressing the prediction of HT TTs in mines using the following query: “TITLE-ABS-KEY ( ( mine OR mines ) AND ( predict OR forecast ) AND ( time OR times OR timing OR duration* OR productivity ) AND ( route* OR course* OR itinerar* OR road* OR track OR tracks OR travel OR travels OR distance* OR haul OR hauls OR cycle* ) AND ( truck* OR hauler* OR ht OR dumper* ) ) AND ( LIMIT-TO ( LANGUAGE, ’English’) ) AND ( EXCLUDE ( SUBJAREA, ’MEDI’) ) AND ( EXCLUDE ( SUBJAREA, ’BIOC’) ) AND ( EXCLUDE ( SUBJAREA, ’AGRI’) ) AND ( EXCLUDE ( SUBJAREA, ’SOCI’) ) AND ( EXCLUDE ( SUBJAREA, ’ENVI’) ) AND ( EXCLUDE ( SUBJAREA, ’ENER’) )”. On April 21, 2023, a search in Scopus using the Compendex and Inspec databases returned 67 documents. Additionally, on October 4, 2023, we searched Google Scholar using the keywords from the previous Scopus query and collected the first 50 documents returned. The successive steps of our systematic identification protocol are illustrated in Fig. 12. Ultimately, it only retained 15 papers. An overview of these 15 documents can be found in Table 5.

**Table 5:** Overview of selected papers

N°	*Title*	Mine type	Prediction method
1	*Weighted ensembles of artificial neural networks based on Gaussian mixture modeling for truck productivity prediction at open-pit mines* [5]	Open-pit	ML
2	*Underground mine truck travel time prediction based on stacking integrated learning* [8]	Underground	ML
3	*Prediction method of truck travel time in open-pit mines based on LSTM model* [15]	Open-pit	ML
4	*Coordinating multiple cooperative vehicle trajectories on shared road networks* [21]	Open-pit	Optimization
5	*Prediction of truck productivity at mine sites using tree-based ensemble models combined with Gaussian mixture modelling* [10]	Open-pit	ML
6	*Preprocessing large datasets using Gaussian mixture modelling to improve prediction accuracy of truck productivity at mine sites* [11]	Open-pit	ML
7	*Use of machine learning algorithm models to optimize the fleet management system in opencast mines* [12]	Open-pit	ML
8	*A simulation model for estimation of mine haulage fleet productivity* [19]	Open-pit	Simulation
9	*Simulation of truck haulage operations in an underground mine using big data from an ICT-based mine safety management system* [17]	Underground	Simulation
10	*The use of a machine learning method to predict the real-time link travel time of open-pit trucks* [7]	Open-pit	ML
11	*Dispatch with confidence: integration of machine learning, optimization and simulation for open-pit mines* [14]	Open-pit	ML
12	*Simulation and optimization in open-pit mining* [18]	Open-pit	Simulation
13	*A comparative study of truck cycle time prediction methods in open-pit mining* [13]	Open-pit	ML & Simulation
14	*Modelling performance and retarder chart of off-highway trucks by cubic splines for cycle time estimation* [20]	Open-pit	Mathematical Model
15	*A computerized model for truck dispatching in open-pit mines* [16]	Open-pit	ML

## Appendix 2: Hyperparameters optimization

**Table 6:** Hyperparameters to optimize and optimal values

	Model	Python resource	Hyperparameters to optimize	Value ranges tested	Mine 1			Mine 2			
					Complete	Short	Major	Conventional			Auto
								Complete	Short	Major	Complete
Clustering	GMM	GaussianMixture.aic from sklearn.mixture	Nb of clusters	$\mathcal {U}\{1, 20\}$	5	4	6	4	3	{6, 6}$^{1}$	2
	MLP	RandomSearch fromkeras_tuner	Nb of units - 1st hidden layer	$\mathcal {U}\{32, 256\}$	192	192	160	64	96	{148, 104}$^{1}$	224
			Nb of units - 2nd hidden layer	$\mathcal {U}\{8, 128\}$	16	8	8	8	8	{21, 30}$^{1}$	16
			Batch size	$\mathcal {U}\{16, 128\}$	48	32	48	128	32	{34, 22}$^{1}$	16
			Learning rate	$\mathcal {U}(1 \times 10^{-4}, 1 \times 10^{-2})$	$1 \times 10^{-3}$	$1 \times 10^{-3}$	$1 \times 10^{-2}$	$1 \times 10^{-2}$	$1 \times 10^{-2}$	{$1 \times 10^{-2}$, $3 \times 10^{-2}$}$^{1}$	$1 \times 10^{-2}$
Initialpredictions	XGBoost	RandomizedSearchCV from sklearn. model_selection	Learning rate	$\mathcal {U}(1 \times 10^{-3}, 1 \times 10^{-2})$	$5 \times 10^{-3}$	$7 \times 10^{-3}$	$1 \times 10^{-2}$	-	-	-	-
			Max depth	$\mathcal {U}\{2, 10\}$	9	6	5	-	-	-	-
			Nb of estimators	$\mathcal {U}\{100, 500\}$	400	364	428	-	-	-	-
			Subsample	$\mathcal {U}(0.5, 0.7)$	0.6	0.5	0.8	-	-	-	-
	RF	RandomizedSearchCV from sklearn. model_selection	Max depth	$\{None, 10, 20, 30, 40, 50\}$	None	10	10	-	-	-	-
			Max features	$\{auto, sqrt\}$	sqrt	log2	sqrt	-	-	-	-
			Min samples leaf	$\mathcal {U}\{1, 10\}$	6	7	1	-	-	-	-
			Min samples split	$\mathcal {U}\{2, 15\}$	10	12	12	-	-	-	-
			Nb of estimators	$\mathcal {U}\{100, 200\}$	150	175	192	-	-	-	-
	GBR	RandomizedSearchCV from sklearn. model_selection	Learning rate	$\mathcal {U}(1 \times 10^{-3}, 5 \times 10^{-2})$	$1 \times 10^{-2}$	$1 \times 10^{-2}$	$2 \times 10^{-2}$	-	-	-	-
			Max depth	$\mathcal {U}\{3, 10\}$	6	4	4	-	-	-	-
			Nb of estimators	$\mathcal {U}\{100, 300\}$	256	196	204	-	-	-	-
			Max features	$\{auto, sqrt, log2\}$	sqrt	log2	sqrt	-	-	-	-
			Min samples leaf	$\mathcal {U}\{1, 10\}$	6	3	2	-	-	-	-
			Min samples split	$\mathcal {U}\{2, 15\}$	10	5	4	-	-	-	-
	SVR	RandomizedSearchCV from sklearn. model_selection	C	$\mathcal {U}\{1, 100\}$	20	95	95	-	-	-	-
			Epsilon	$\mathcal {U}(1 \times 10^{-2}, 1 \times 10^{-1})$	$5 \times 10^{-2}$	$1 \times 10^{-1}$	$1 \times 10^{-1}$	-	-	-	-
			Kernel	$\{linear, poly, rbf, sigm\}$	rbf	linear	linear	-	-	-	-
	BRNN	RandomSearch fromkeras_tuner	Nb of units - 1st hidden layer	$\mathcal {U}\{32, 512\}$	256	32	128	-	-	-	-
			L2 - 1st hidden layer	$\mathcal {U}(1 \times 10^{-4}, 1 \times 10^{-1})$	$3 \times 10^{-3}$	$1 \times 10^{-3}$	$3 \times 10^{-2}$	-	-	-	-
			Nb of units - 2nd hidden layer	$\mathcal {U}\{4, 128\}$	20	32	28	-	-	-	-
			L2 - 2nd hidden layer	$\mathcal {U}(1 \times 10^{-4}, 1 \times 10^{-2})$	$3 \times 10^{-3}$	$1 \times 10^{-3}$	$1 \times 10^{-2}$	-	-	-	-
			Batch size	$\mathcal {U}\{16, 256\}$	128	160	480	-	-	-	-
			Learning rate	$\mathcal {U}(1 \times 10^{-4}, 1 \times 10^{-1})$	$1 \times 10^{-3}$	$1 \times 10^{-1}$	$1 \times 10^{-1}$	-	-	-	-
	LSTM	RandomSearch fromkeras_tuner	Nb of units - 1st hidden layer	$\mathcal {U}\{16, 512\}$	48	32	272	244	333	{142, 410}$^{1}$	174
			L2 - 1st hidden layer	$\mathcal {U}(1 \times 10^{-4}, 1 \times 10^{-2})$	$3 \times 10^{-3}$	$1 \times 10^{-3}$	$4 \times 10^{-3}$	$9 \times 10^{-3}$	$7 \times 10^{-3}$	{$4 \times 10^{-4}$, $4 \times 10^{-3}$}$^{1}$	$4 \times 10^{-4}$
			L2 - output layer	$\mathcal {U}(1 \times 10^{-4}, 1 \times 10^{-1})$	$1 \times 10^{-4}$	$1 \times 10^{-3}$	$1 \times 10^{-1}$	$2 \times 10^{-1}$	$1 \times 10^{-1}$	{$6 \times 10^{-2}$, $1 \times 10^{-1}$}$^{1}$	$6 \times 10^{-2}$
			Batch size	$\mathcal {U}\{32, 512\}$	416	408	408	109	218	{480, 25}$^{1}$	33
			Learning rate	$\mathcal {U}(1 \times 10^{-4}, 1 \times 10^{-2})$	$3 \times 10^{-3}$	$5 \times 10^{-4}$	$1 \times 10^{-3}$	$2 \times 10^{-3}$	$1 \times 10^{-3}$	{$8 \times 10^{-4}$, $1 \times 10^{-3}$}$^{1}$	$2 \times 10^{-3}$
Postprocessing for segmentedroutes only	Residual regression	-	-	-	-	-	-	-	-	-	-
	MLR	Manual iterations	$\lambda _{\text {reg}}$	$\mathcal {U}(1 \times 10^{-5}, 1 \times 10^{5})$	-	$1 \times 10^{4}$	$1 \times 10^{4}$	-	$1 \times 10^{4}$	$1 \times 10^{4}$	-
Stacking	Linear regression	optuna $^{2}$	Fit intercept	$\{True, False\}$	-	-	-	*False*			-
	GBR		Nb of estimators	$\mathcal {U}\{50, 500\}$	445			-	-	-	-
			Max depth	$\mathcal {U}\{2, 5\}$	2			-	-	-	-
			Learning rate	$\mathcal {U}(1 \times 10^{-3}, 1 \times 10^{-1})$	$1.35 \times 10^{-2}$			-	-	-	-
			Subsample	$\mathcal {U}(0.5, 1)$	0.8			-	-	-	-
	RF		Max depth	$\mathcal {U}\{2, 5\}$	-	-	-	-	-	-	-
			Max features	$\{None, sqrt, log2\}$	-	-	-	-	-	-	-
			Nb of estimators	$\mathcal {U}\{50, 500\}$	-	-	-	-	-	-	-
	SVR		C	$\mathcal {U}(0.1, 100)$	-	-	-	-	-	-	-
			Epsilon	$\mathcal {U}(1 \times 10^{-2}, 1)$	-	-	-	-	-	-	-
			Kernel	$\{linear, poly, rbf, sigm\}$	-	-	-	-	-	-	-

$^{1}$ There are two major segments in Mine 2

$^{2}$ For further details about this library, see Optuna

## References

[1] Mitchell P (2024) Top 10 mining and metals risks in 2025. Technical report, EY. https://www.ey.com/en_gl/insights/energy-resources/risks-opportunities Accessed 2024-11-24

[2] McBrayer A, Brickey A (2023) A review of current scheduling and design practices in the powder river basin. Min Metall Explor 40(4):1059–1080. 10.1007/s42461-023-00754-w

[3] Michelle B, Adrian RP, Peter JS (2017) Short-term scheduling of an open-pit mine with multiple objectives. Eng Optim 49(5):777–795. 10.1080/0305215X.2016.1218002

[4] L’Heureux G, Gamache M, Soumis F (2013) Mixed integer programming model for short term planning in open-pit mines. Min Technol 122:101–109. 10.1179/1743286313Y.0000000037

[5] Fan C, Zhang N, Jiang B, Liu WV (2023) Weighted ensembles of artificial neural networks based on gaussian mixture modeling for truck productivity prediction at open-pit mines. Min Metall Explor 40(2):583–598. 10.1007/s42461-023-00747-9

[6] Campeau L-P, Gamache M (2019) Short-term planning optimization model for underground mines. Comput Oper Res 115:(2019). 10.1016/j.cor.2019.02.005

[7] Sun X, Zhang H, Tian F, Yang L (2018) The use of a machine learning method to predict the real-time link travel time of open-pit trucks. Math Prob Eng. 10.1155/2018/4368045

[8] Li N, Wu Y, Wang Q, Ye H, Wang L, Jia M, Zhao S (2023) Underground mine truck travel time prediction based on stacking integrated learning. Eng Appl Artif Intell 120:105873. 10.1016/j.engappai.2023.105873

[9] Zare M, Battulwar R, Seamons J, Sattarvand J (2021) Applications of wireless indoor positioning systems and technologies in underground mining: a review. Min Metall Explor 38(6):2307–2322. 10.1007/s42461-021-00476-x

[10] Fan C, Zhang N, Jiang B, Liu WV (2022) Prediction of truck productivity at mine sites using tree-based ensemble models combined with gaussian mixture modelling. Int J Min Reclam Environ 37(1):66–86. 10.1080/17480930.2022.2142425

[11] Fan C, Zhang N, Jiang B, Liu WV (2022) Preprocessing large datasets using Gaussian mixture modelling to improve prediction accuracy of truck productivity at mine sites. Arch Min Sci 67(4):661– 680. 10.24425/ams.2022.143680

[12] Choudhury S, Naik H (2022) Use of machine learning algorithm models to optimize the fleet management system in opencast mines. Int J Innov Res Sci Eng Technol 7:884–892. 10.5281/zenodo.6812563

[13] Chanda E, Gardiner S (2010) A comparative study of truck cycle time prediction methods in open-pit mining. Eng Constr Archit Manag 17:446–460. 10.1108/09699981011074556

[14] Ristovski K, Gupta C, Harada K, Tang H-K (2017) Dispatch with confidence: Integration of machine learning, optimization and simulation for open pit mines. In: Proceedings of the 23rd ACM SIGKDD International Conference on Knowledge Discovery and Data Mining, pp 1981– 1989. Association for Computing Machinery, New York, NY, USA. 10.1145/3097983.3098178

[15] Ao M, Li C, Yang S (2023) Prediction method of truck travel time in open pit mines based on LSTM model. In: 2023 42nd Chinese Control Conference (CCC), pp 8651– 8656. 10.23919/CCC58697.2023.10240705

[16] Otuonye FO, Temeng VA (1997) A computerized model for truck dispatching in open pit mines. Eng Environ Sci. https://www.proquest.com/dissertations-theses/computerized-modeltruck-dispatching-open-pit/docview/304383141/se-2?accountid=40695

[17] Baek J, Choi Y (2019) Simulation of truck haulage operations in an underground mine using big data from an ICT-based mine safety management system. Appl Sci 9:(13). 10.3390/app9132639

[18] Upadhyay SP, Askari-Nasab H, Tabesh M, Badiozamani MM (2015) Simulation and optimization in open pit mining. In: Applications of Computers and Operations Research in Mineral Industry - 37th APCOM. https://www.researchgate.net/publication/343682343_Simulation_and_Optimization_in_Open_Pit_Mining#fullTextFileContent

[19] Upadhyay S, Tabesh M, Badiozamani M, Askari-Nasab H (2020)A simulation model for estimation of mine haulage fleet productivity. In: Topal E (ed) Proceedings of the 28th International Symposium on Mine Planning and Equipment Selection - MPES 2019, pp 42– 50. Springer, Cham. 10.1007/978-3-030-33954-8_5

[20] Erarslan K (2005) Modelling performance and retarder chart of off-highway trucks by cubic splines for cycle time estimation. Min Technol 114(3):161–166. 10.1179/037178405X54006

[21] Gun P, Hill AJ, Vujanic R (2023) Coordinating multiple cooperative vehicle trajectories on shared road networks. IEEE Trans Intell Transp Syst 24(1):274–290. 10.1109/TITS.2022.3215573

[22] Scientist WF data: one-hot encoding — a brief explanation(2023) https://medium.com/@WojtekFulmyk/one-hot-encoding-a-brief-explanation-8c5daec395e3 Accessed 2024-08-07

[23] McLachlan G, Peel D (2000) Finite mixture models. John Wiley & Sons. 10.1002/0471721182

[24] Haykin S (1998) Neural networks: a comprehensive foundation, 2nd edn. Prentice Hall PTR. 10.5555/521706

[25] Breiman L (2001) Random forests. Mach Learn 45(1):5–32. 10.1023/A:1010933404324

[26] Chen T, Guestrin C (2016) Xgboost: a scalable tree boosting system. In: Proceedings of the 22nd ACM SIGKDD International Conference on Knowledge Discovery and Data Mining, pp 785– 794. 10.1145/2939672.2939785. ACM

[27] Friedman JH (2001) Greedy function approximation: a gradient boosting machine. Ann Stat 29(5):1189–1232. 10.1214/aos/1013203451

[28] Cortes C, Vapnik V (1995) Support-vector networks. Mach Learn 20(3):273–297. 10.1007/BF00994018

[29] Fortunato M, Blundell C, Vinyals O (2019) Bayesian recurrent neural networks. 10.48550/arXiv.1704.02798

[30] Hochreiter S, Schmidhuber J (1997) Long short-term memory. Neural Comput 9(8):1735–1780. 10.1162/neco.1997.9.8.1735 9377276 10.1162/neco.1997.9.8.1735

[31] James G, Witten D, Hastie T, Tibshirani R, Taylor J (2023) An introduction to statistical learning with applications in Python. Springer. 10.1007/978-3-031-38747-0

[32] Seber GA, Lee AJ (2012) Linear regression analysis. John Wiley & Sons. 10.1002/9780471722199

[33] Bischl B, Binder M, Lang M, Pielok T, Richter J, Coors S, Thomas J, Ullmann T, Becker M, Boulesteix A-L, Deng D, Lindauer M (2023) Hyperparameter optimization: foundations, algorithms, best practices, and open challenges. WIREs Data Mining Knowl Discov 13(2):1484. 10.1002/widm.1484

[34] Akaike H (1974) A new look at the statistical model identification. IEEE Trans Autom Control 19(6):716–723. 10.1109/TAC.1974.1100705

[35] Hyndman RJ, Athanasopoulos G (2021) Forecasting: principles and practice, 3rd edn. OTexts: Melbourne, Australia. https://otexts.com/fpp3/

[36] Akiba T, Sano S, Yanase T, Ohta T, Koyama M (2019) Optuna: a next-generation hyperparameter optimization framework. In: KDD ’19, pp 2623– 2631. Association for Computing Machinery, New York, NY, USA. 10.1145/3292500.3330701

